# The Effect of Fearful Expressions on Multiple Face Tracking

**DOI:** 10.5334/pb.bi

**Published:** 2015-07-09

**Authors:** Hongjun Jin, Baihua Xu

**Affiliations:** 1 Department of Psychology and Behavioral Sciences, Zhejiang University, China

**Keywords:** Fearful expression, Multiple face tracking, Attention, High-level social cognition

## Abstract

How does the visual system realize dynamic tracking? This topic has become popular within cognitive science in recent years. The classical theory argues that multiple object tracking is accomplished via pre-attention visual indexes as part of a cognitively impenetrable low-level visual system. The present research aimed to investigate whether and how tracking processes are influenced by facial expressions that convey abundant social information about one’s mental state and situated environment. The results showed that participants tracked fearful faces more effectively than neutral faces. However, this advantage was only present under the low-attentional load condition, and distractor face emotion did not impact tracking performance. These findings imply that visual tracking is not driven entirely by low-level vision and encapsulated by high-level representations; rather, that facial expressions, a kind of social information, are able to influence dynamic tracking. Furthermore, the effect of fearful expressions on multiple face tracking is mediated by the availability of attentional resources.

## Introduction

Many daily activities require people to track several objects at the same time. For instance, drivers need to constantly keep track of the movements of other cars in order to avoid collisions. Also, when an athlete is playing basketball, he/she needs to keep an eye on the changing positions of his or her teammates and opponents in order to make effective decisions related to whether to attack or defend. Tracking ability is therefore a must-have survival skill for safer, better lives.

**Figure 1:**
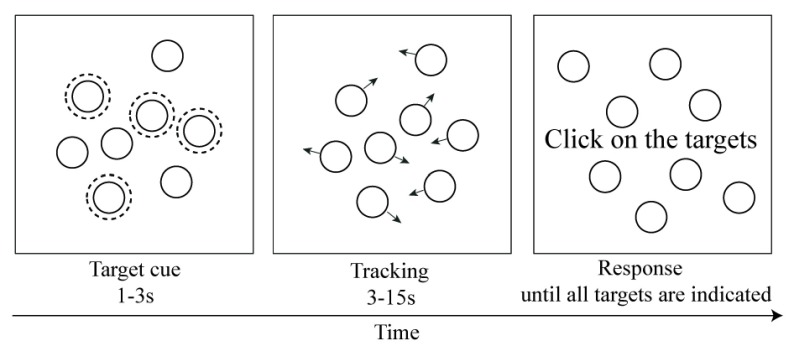
A typical MOT trial. At the start of the trial, four identical objects flash in order to indicate that they are targets to be tracked. All objects then begin moving around the display. At end of the trial, the participant is required to click on the four targets.

Pylyshyn and Storm (1988) pioneered the investigation into the dynamic tracking ability of our visual system under the lens of the multiple object tracking (MOT) paradigm. In a typical MOT task, participants are presented with a field of identical objects, some of which are cued as targets to be tracked. All objects then move independently and randomly for a period of time, after which participants are asked to identify all the targets (see Figure 1).

Based on the MOT paradigm, Pylyshyn (1989, 2001, 2007) further proposed the visual index theory to explain people’s visual tracking mechanism. This theory argues that MOT is executed by an early or low-level visual system, and that this system can offer four to five indexes that may be assigned to visual objects. Here, an index is considered to be a reference token, or a point to connect a person’s visual system with the real physical world. As such, an index only conveys the location information of objects, but does not encode or represent any feature information of objects (i.e., in that sense it is feature-blind; Pylyshyn, 1989, 1994, 2007). Once designated to a target, the index will always stick to the target no matter how the target moves, and if the number of targets falls within the maximum capacity of the early visual system, then tracking will be successful. According to Pylyshyn’s theory, index maintenance is automatically carried out by the early visual system without any attentional effort, and the whole tracking process is achieved without any involvement of high-level cognition, although it may require a specific memory subsystem for its own operation.

In Pylyshyn and Storm’s (1988) original MOT task, participants are asked to discriminate between tracked targets and untracked distractors and tested for their ability to retrieve target locations. Since the moving objects in MOT studies are typically identical, tracking is achieved primarily by updating objects’ spatiotemporal information and overlooks any contribution from feature or identity information. However, in reality, tracked objects usually have distinct identities and people do not only need to track the positions of multiple objects (i.e., “where” information), but also the content of a particular target (i.e., “what” information). As a target is continuously moving, observers need to continuously bind the target’s identity with its new location. On the basis of preceding MOT studies, researchers developed a multiple identity tracking (MIT, Oksama & Hyönä, 2004, 2008) paradigm in order to afford identity information to objects and to explore the cognitive processing mechanisms of dynamic tracking in the real world. MIT is commonly represented by a tracking task in which each target has a distinct identity. According to the way in which responses are made, there are two forms of MIT task: one requires observers to report the targets’ locations, but to ignore their identities, and thus investigates whether people are able to use feature information to aid tracking; whereas the other requires observers to report both the locations and identities of targets, and thus investigates whether people are able to track the identities of distinct objects.

A host of MIT research has shown that identity information can be processed and influence tracking capability, suggesting the presence of a content-addressable representation during tracking (knowing which target is where; Horowitz, Klieger, Fencsik, Yang, Alvarez, & Wolfe, 2007; Howe & Holcombe, 2012; Makovski & Jiang, 2009a; Makovski & Jiang, 2009b). The content-addressable representation, however, has a lower capacity than the location-addressable representation (knowing where the targets are; Botterill, Allen, & McGeorge, 2011; Cohen, Pinto, Howe, & Horowitz, 2011). Furthermore, the capacity of observers to successfully identify and track targets depends on feature types (Liu, Chen, Liu, & Fu, 2012; Liu, Chen, Xuan, & Fu, 2009).

In early research, simple physical features such as color, shape, size and line direction, were often used as identity features. However, objects in the real world not only contain physical properties, but also hold social properties. Apparently, processing the social information of an object is more complex than processing physical features alone. As complex visual stimuli, faces contain a large amount of social information, including gender, identity, emotion, personality and ethnicity. In recent years, researchers have started to explore how faces are tracked (Allen & Gabbert, 2013; Liu & Chen, 2012; Oksama & Hyönä, 2008; Ren, Chen, Liu, & Fu, 2009). Oksama and Hyönä (2008) firstly employed faces as tracking stimuli in an MIT experiment that investigated the effects of facial familiarity on identity tracking. They found that pseudo-faces, created from famous faces (e.g., Albert Einstein and Bill Clinton) by deconstructing them and then rearranging and recombining the parts, were harder to track than famous faces. In another study, Ren and colleagues (2009) focused on whether identity processing of unfamiliar faces is mandatory without deliberate intentions. They showed that target facial identities are processed during tracking even when such encoding is irrelevant to the task. This mandatory identity processing was shown to interfere with tracking capability, as it competed with this process for limited attentional resources (Ren, Chen, Liu, & Fu, 2009). Researchers further found that attentional tracking can be biased by contextual information about the target face’s social roles (Allen & Gabbert, 2013) and the attractiveness of target faces (Liu & Chen, 2012).

Facial expressions also play an important role in facial studies, as these reflect an individual’s mental state and convey abundant social information related to the situated environment. Effective recognition of facial expressions helps individuals succeed in social interactions (Van Kleef, 2009). Taylor and Therrien (2005) further argue that facial expressions influence the attentional bias of observers, negative facial expressions (especially fear) can quickly capture our attention (Carlson & Reinke, 2010; Carlson, Reinke, & Habib, 2009; Eastwood, Smilek, & Merikle, 2001, 2003; Eimer & Kiss, 2007; Fox, 2002; Globisch, Hamm, Esteves, & Öhman, 1999; Pourtois, Grandjean, Sander, & Vuilleumier, 2004).

The need to track multiple moving faces, each of which convey facial expressions, is quite a frequent occurrence in everyday life. For instance, a kindergarten teacher needs to be able to simultaneously care for numerous children, each demonstrating different emotions through expression. It is surprising then that no research has ever explored the effects of facial expressions on attentional tracking in which sustained attention is distributed among multiple moving faces. Previous studies related to the influence of facial expressions on attention have only adopted a static paradigm, and, while they have demonstrated that responses to faces with negative facial expressions are rapid and transient, it remains unknown whether such an attentional bias only exists for a brief period. Therefore, the present study aimed to explore whether there is a similar effect of facial expressions on attentional tracking. In particular, recognition of fearful emotions conveys useful information for individuals’ survival, as efficient detection helps individuals avoid potential dangers in the environment (Masterson & Crawford, 1982; Mineka & Öhman, 2002). Considering the ecological significance of fearful emotions, the present research employed fearful faces as tracking stimuli.

In addition to the above, researchers have not yet reached a consensus on whether facial expression processing acts independently from attentional modulation. In other words, is facial expression processing automatic or controlled? Automatic processing would imply that facial expression processing does not require attentional resources and is not influenced by cognitive control (Anderson, Christoff, Panitz, De Rosa, & Gabrieli, 2003; Esteves, Dimberg, & Öhman, 1994; Öhman, 2002; Vuilleumier, Armony, Driver, & Dolan, 2001), while in contrast, the controlled view suggests that the processing of facial expressions is influenced by the extent of available attentional resources (Eimer, Holmes, & McGlone, 2003; Holmes, Vuilleumier, & Eimer, 2003; Pessoa, McKenna, Gutierrez, & Ungerleider, 2002; Pessoa, Padmala, & Morland, 2005). Another aim of the present study was therefore to examine whether attentional resources mediate any effect of fearful expressions on multiple face tracking.

We manipulated the attentional load of tracking by changing the relative proximity of objects to each other. Previous research has found that proximity is the root cause of all performance constraints in visual tracking (Bae & Flombaum, 2012; Franconeri, Jonathan, & Scimeca, 2010; Franconeri, Lin, Enns, Pylyshyn, & Fisher, 2008; Iordanescu, Grabowecky, & Suzuki, 2009; Shim, Alvarez, & Jiang, 2008), as decreased distance between objects requires more precise object representation, and the limited spatial resolution of attention does not meet this requirement. Hence, more attentional resources are required to distinguish between targets and distractors. Increases in object speed, trial duration, target load and number of distractors, all increase the frequency with which targets and distractors are in close proximity, and thus indirectly impair tracking performance. In this study, we adopted the planets and moons tracking (PMT) paradigm, proposed by Tombu and Seiffert (2011), in which object speed and proximity can be independently manipulated. The motion pattern during PMT is similar to the motion of planets and moons as each target-distractor group rotates around their group center in addition to the screen center.

The present research hypothesized that if the processing of fearful facial expressions is automatic, then target faces with fearful expressions would improve tracking performance, while distractor faces with fearful expressions would harm tracking performance. In contrast, if the processing of fearful facial expressions is modulated by attentional load, then this effect would be expected to be present only under the low-attentional load condition.

## Method

### Ethics Statement

The study was approved by the Research Ethics Board of Zhejiang University and all participants provided written informed consent before taking part in the experiment.

### Participants

A total of 19 undergraduate students (13 males) with normal or corrected-to-normal vision took part in this study in exchange for course credits or monetary payment. The age of the participants ranged from 18 to 27 years (M = 22.01, SD = 2.89).

### Stimuli and Apparatus

Facial images were selected from photographs of 39 different individuals (23 men and 16 women) taken from the MacBrain Face Stimulus Set (http://www.macbrain.org)^1^. Fearful and neutral expressions were selected for each face, resulting in a total of 78 facial pictures. Each picture was processed to exclude all features except the eyes, eyebrows, nose and mouth. All images were scaled to the same size: 2.5° in length and 2.2° in width, and were further manipulated to the same mean luminance and root-mean-square contrast.

Participants were tested individually in a room with normal interior lighting and sat approximately 57 cm away from a 19-inch CRT monitor with a pixel resolution of 1,600 × 1,200 and a refresh rate of 85 Hz. The background color of the display was black. The experimental procedure was generated in psychopy (Peirce, 2007, 2008).

### Procedure

**Figure 2:**
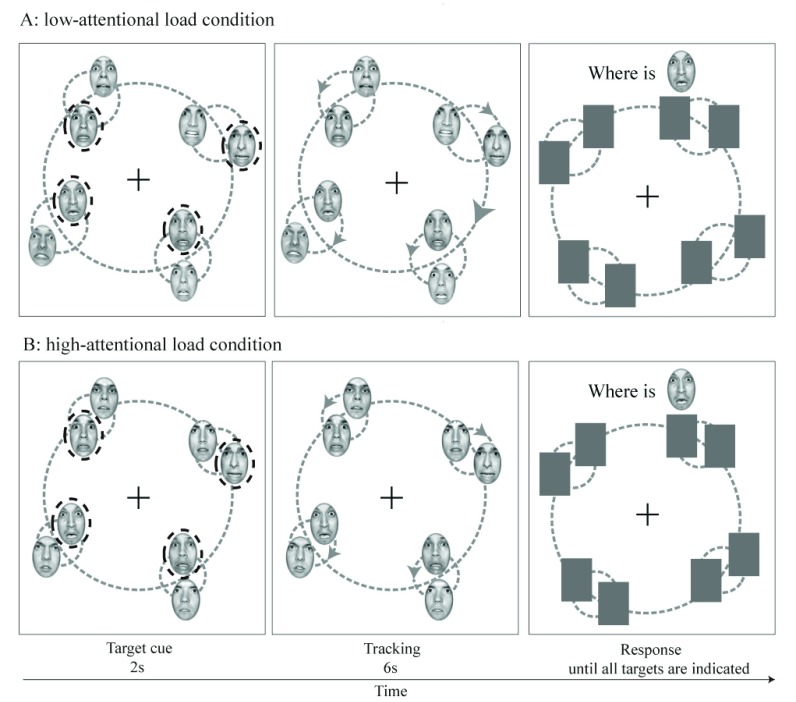
Illustration of the trial procedure used in our study. **Figure 2A** shows an example of tracking fearful target faces among fearful distractor faces in the low-attentional load condition, while **Figure 2B** shows an example of tracking fearful target faces among neutral distractor faces in the high-attentional load condition. During the target cue phase, four groups of faces were arranged evenly along a large imaginary circle. Each group consisted of one target face and one distractor face, which were in turn distributed evenly on a small imaginary circle. The target faces to be tracked were flashed three times in order to highlight them to the participant. The target cues then disappeared and all faces began rotating. Two face groups rotated clockwise around their group center, and the other two rotated counter-clockwise. Each group simultaneously rotated around the screen center. During the response phase, all faces were occluded by grey rectangles. Participants were then shown each target face in turn and asked to click on its location.

On all trials, participants were asked to track four target faces among four distractor faces. The eight faces presented in each trial were of the same sex. They were randomly chosen for each trial from the facial database. Each trial comprised of a target cue phase, a tracking phase and a response phase. The procedure in each trial is illustrated in Figure 2.

During the target cue phase, four groups of faces were located equidistantly along a large imaginary circle (radius = 7.5°) centered on a white fixation cross (1.0° × 1.0°) at the screen center. Each group consisted of two faces, one of which flashed on and off three times over a two-second period in order to identify it as a target. The other faces remained constantly visible during this time. Each group therefore consisted of a target face and a distractor face that were distributed equidistantly on a small imaginary circle whose perimeter passed through the center of each face. Two conditions of distance were created based on the different radii of the small imaginary circle, and thus two attentional loads were generated. The distance between target and distractor faces varied from 3.6° (high-attentional load) to 4.4° (low-attentional load) of visual angle. The selection of these two distances was based on the critical distance of 4° of visual angle that has been used in previous studies (Bae & Flombaum, 2012; Franconeri, Alvarez, & Enns, 2007). The distance was fixed within trials, but varied across trials.

At the start of the tracking phase, target cues disappeared and all faces moved around the screen for six seconds. In half of the trials, face groups rotated clockwise around the fixation cross and rotated counter-clockwise in the other half. Both rotated at a speed of 60°/s. On each trial, two face groups rotated clockwise around their group center, while the other two rotated counter-clockwise. The angular speed at which the faces rotated around the center of their group was 90°/s and 73°/s for the near and far distance conditions respectively. This manipulation ensured that all faces traveled an equal linear distance across all the experimental distance conditions.

At the end of the tracking period, all eight faces stopped moving and were occluded by grey rectangles, whose size was equal to the faces (response phase). Thereafter, a series of four questions were presented in the upper section of the screen, one at a time, in order to test for each target face. Questions were of the form “Where is ____” with the blank to be filled by the image of a specific target face. Participants were instructed to click on each specific target face in turn. No feedback was given and the spacebar was used to initiate the next trial.

Participants were instructed to keep their eyes focused on the fixation cross throughout the whole experiment, but eye movements were not monitored as they have been shown not to affect tracking performance. Pylyshyn and Storm (1988) monitored fixation and eliminated trials on which participants made eye movements. Under these conditions, they obtained qualitatively similar results to other studies that either required participants to maintain fixation but did not monitor eye movements (e.g., Allen, Mcgeorge, Pearson, & Milne, 2006; Scholl & Pylyshyn, 1999; Sears & Pylyshyn, 2000), or else employed no special instructions concerning fixation (e.g., Intriligator & Cavanagh, 2001; Scholl, Pylyshyn, & Feldman, 2001; Yantis, 1992).

### Design

We employed a 2 (expression of target faces: fearful vs. neutral) × 2 (expression of distractor faces: fearful vs. neutral) × 2 (attentional load: low vs. high) within-subjects design. Target faces, which were either all fearful or all neutral, were tracked among distractor faces, which were also either all fearful or all neutral. Attentional load was manipulated by varying the distance between target and distractor faces. In the low-attentional load condition, target and distractor faces were relatively far away from each other and so little attentional resources were needed to distinguish between the two. In contrast, in the high-attentional load condition, target and distractor faces were relatively near to each other, and so distractor faces would sometimes become confused with target faces and therefore demand a much greater allocation of attentional resources in order to distinguish between target and distractor faces.

Each of the eight conditions included 20 trials, and all 160 trials were presented in one block in random order. The experiment began with eight practice trials followed by 160 test trials, and took about 60 minutes to complete.

### Data analysis

Since we were primarily concerned with whether or not participants could successfully track the target faces, only accuracy was assessed, as response latencies were not very informative about performance.

Location and identity tracking accuracies were included in the present analysis. Location tracking accuracy refers to participants’ ability to differentiate between tracked targets and untracked distractors, whereas identity tracking accuracy refers to their ability to know which target is where. Imagine that a participant was tracking four faces, whose names were Tom, Jack, Tony and Alex. If the participant was required to click on Tom, and instead clicked on Jack, this would be considered a hit with respect to location tracking accuracy, because it indicated that the participant knew it was a target, but as a miss with respect to identity tracking accuracy, because the participant did not know which target it was. According to the above definition, identity tracking accuracy can never exceed location tracking accuracy (Allen & Gabbert, 2013; Cohen et al., 2011; Horowitz et al., 2007; Pinto, Howe, Cohen, & Horowitz, 2010). Accuracy was computed by measuring the percentage of faces that were correctly located or identified.

## Results

**Figure 3:**
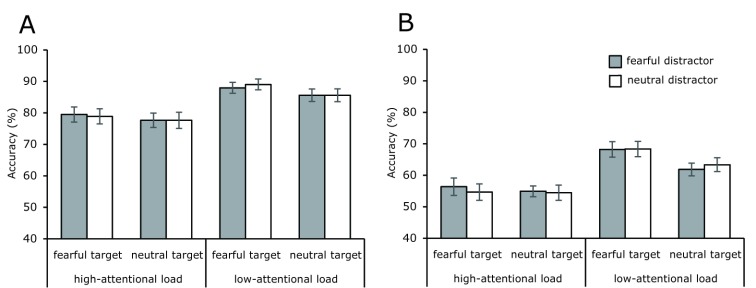
Mean tracking accuracy in low- and high-attentional load conditions as a function of target and distractor facial expressions. (A) Results for location tracking. (B) Results for identity tracking. Error bars indicated ±1 standard errors.

Location and identity tracking accuracies are plotted in Figure 3. An alpha level of .05 was used for all statistical analyses in this study. A four-way within-subjects repeated measures ANOVA was performed, with the expression of target faces, the expression of distractor faces, the attentional load and the task (location versus identity) as factors.

Results indicated the presence of a significant main effect of task so that location accuracy was significantly greater than identity accuracy, *F* (1, 18) = 94.473, *p* < .001, ŋ_p_^2^ = 0.840. There was also a significant main effect of attentional load so that accuracy was higher when the attentional load was lower, *F* (1, 18) = 124.576, *p* < .001, ŋ_p_^2^ = 0.874. This finding is consistent with those of previous studies (Bae & Flombaum, 2012; Franconeri, Jonathan, & Scimeca, 2010; Franconeri, Lin, Enns, Pylyshyn, & Fisher, 2008; Iordanescu, Grabowecky, & Suzuki, 2009; Makovski & Jiang, 2009b; Shim, Alvarez, & Jiang, 2008). The main effect of target facial expressions was also significant so that accuracy was higher for fearful, rather than neutral target faces, *F* (1, 18) = 17.152, *p* = .001, ŋ_p_^2^ = 0.488. However, no significant main effect was found for distractor facial expressions, *F* (1, 18) = 0.039, *p* = .845, ŋ_p_^2^ = 0.002. Most importantly, there was a significant interaction between attention al load and expression of target faces, *F* (1, 18) = 9.837, *p* = .006, ŋ_p_^2^ = 0.353. A simple effect test confirmed that the accuracy difference between fearful and neutral target faces was only significant in the low-attentional load condition, *F* (1, 18) = 21.938, *p* < .001, ŋ_p_^2^ = 0.549, and was eliminated in the high-attentional load condition, *F* (1, 18) = 1.212, *p* = .285, ŋ_p_^2^ = 0.063. None of the other two-way, three-way or four-way interactions was significant, *ps* > .05.

## Discussion

The present research manipulated the expressions of both target and distractor faces and attentional load in order to explore the effect of fearful expressions on multiple face tracking. Results indicated that target facial expressions influenced tracking performance within the low-attentional load condition so that both location and identity tracking performance improved when the target wore a fearful expression, as opposed to a neutral expression. No difference in tracking performance was found between fearful and neutral target faces in the high-attentional load condition. In contrast, the emotion displayed by distractor faces did not influence tracking performance.

Prior studies have disputed whether or not the processing of emotional faces is mediated by attention. Some researchers suggest that facial expressions are processed automatically and can occur on an unconscious level (Anderson et al., 2003; Dolan & Vuilleumier, 2003; Esteves et al., 1994; Öhman, 2002; Vuilleumier et al., 2001). The most direct evidence supporting this view of automaticity comes from a functional magnetic resonance imaging (fMRI) study in which neural responses to emotional faces across different attention conditions were compared (Vuilleumier et al., 2001). The results of this study revealed that the presence of a significant amygdala response to fearful faces relative to neutral faces, regardless of whether faces were presented inside or outside of the focus of attention. Thus, Vuilleumier et al. (2001) proposed that the processing of fearful faces acts independently from attentional modulation. This postulation is further supported by a similar paradigm created by Anderson et al. (2003), in which participants were asked to pay attention to either houses or faces presented in a single overlapping display. Other researchers propose that the processing of facial expressions is a controlled procedure mediated by available attentional resources (Eimer, Holmes, & McGlone, 2003; Holmes, Vuilleumier, & Eimer, 2003; Pessoa, McKenna, Gutierrez, & Ungerleider, 2002; Pessoa, Padmala, & Morland, 2005). Using fMRI, Pessoa and colleagues (2002) found that all brain regions responding differentially to emotional faces relative to neutral faces, including the amygdala, were activated only when there were sufficient attentional resources to process those faces. When a competing task exhausted all attentional resources, differential responses to emotional faces were shown to vanish. A similar finding was also shown in an event-related potential (ERP) study (Holmes et al., 2003). Furthermore, Pessoa et al. (2005) manipulated attentional load by varying the difficulty of the competing task and found that the presentation of fearful faces without attention resulted in a stronger amygdala response than neutral faces only in the low-attentional demand condition, but not in medium- or high-attentional demand conditions. The authors therefore argue that the processing of task-irrelevant facial expressions depends on the extent to which the processing of task-relevant information leaves redundant resource capacity.

The present findings support the controlled processing view. In the current study, because participants were not required to process facial expressions, fearful faces were not related to the tracking task. However, fearful target faces were found to have a non-ignorable impact on tracking performance, despite the fact that beneficial effects only occurred when the attentional load was low. Specifically, under the low-attentional load condition, target and distractor faces were relatively far away from each other and so fewer attentional resources were needed in order to distinguish one from the other. The remaining attentional resources were therefore able to prioritize the processing of fearful faces and thus strengthened the binding between identity and location, leading to a better tracking performance. Under the high-attentional load condition, target and distractor faces were relatively near to each other and so, most probably, distractor faces may have been mistaken for target faces. In order to maintain successful target tracking, all attentional resources were used to distinguish between target and distractor faces, thus leaving insufficient resources to process facial expressions. This explanation satisfies the finding that there was an absence of an expression effect on tracking under the high-attentional load condition.

Consistent with previous findings, the present research also shows that the targets are able to achieve content-addressable representations during the tracking process (Horowitz et al., 2007; Huang, Zhang, & Zhang, 2014; Liu et al, 2012; Liu et al., 2009; Makovski & Jiang, 2009a; Makovski & Jiang, 2009b; Oksama & Hyönä, 2004, 2008). However, these findings act to discredit the visual index theory proposed by Pylyshyn (1989, 2001, 2007), as this theory argues that visual tracking is controlled by low-level visual mechanisms. Under this conceptualization, the feature information of objects is therefore ignored and inaccessible from higher-level cognition, as visual indexes alone are thought to help realize tracking. However, if target facial expressions are not processed, tracking performance should not be influenced. This was not the case in this study. In contrast to visual index theory, more and more studies have found that high-level cognitive processes can penetrate and influence low-level visual perception (Allen & Gabbert, 2013; de-Wit, Lefevre, Kentridge, Rees, & Saygin, 2011; Liu & Chen, 2012; Oksama & Hyönä, 2008; Ren et al., 2009). The ecological meaning of stimuli, which require high-level representation and include factors such as identity, familiarity, attractiveness and the social label attached to faces, have an effect on tracking performance. The present results further support this argument by suggesting that facial expressions also influence tracking performance. Fearful facial expressions capture individuals’ attention more efficiently and help individuals successfully maintain their targets. Meanwhile, it is also possible that the observed advantage in the tracking of fearful faces is attributable to the low-level visual properties (e.g., luminance, contrast, orientation and spatial frequency) afforded to fearful faces, as opposed to their emotional content per se (Frischen, Eastwood, & Smilek, 2008; Gray, Adams, Hedger, Newton, & Garner, 2013; Purcell, Stewart, & Skov, 1996; Yang & Blake, 2012). Although we carefully scaled all our stimuli to the same size, luminance and contrast, we still cannot rule out the contribution of low-level image differences (at the level of orientation and spatial frequency) between fearful and neutral faces. To clarify this issue, we conducted a control experiment (see Appendix for details of method and results) in which inverted faces were employed as tracking stimuli, while all other parameters remained the same. Under these conditions, fearful faces did not enjoy any tracking advantage over neutral faces, thus indicating that facial expressions influence visual tracking at a high computational level.

In order to achieve successful identity tracking, the visual system must recognize and maintain the representations of multiple face identities. As tracked targets move, these representations should be continuously bound to the corresponding locations (Pinto et al., 2010). Previous research has shown visual working memory is responsible for retaining identity information (Makovski & Jiang, 2009b) and updating identity-location bindings (Oksama & Hyönä, 2008), indicating that visual working memory is closely associated with visual tracking (Allen, Mcgeorge, Pearson, & Milne, 2006; Drew & Vogel, 2008; Fougnie & Marois, 2006, 2009; Howe, Horowitz, Morocz, Wolfe, & Livingstone, 2009; Trick, Mutreja, & Hunt, 2012). Two possibilities may account for the effect of fearful expressions on multiple face tracking in visual working memory. First, the identities of fearful faces may be more effectively retained than those of neutral faces by visual working memory. Indeed, there is a visual working memory maintenance advantage for faces with negative expressions (i.e. angry and fearful faces) relative to those with neutral expressions (Bankó, Gál, & Vidnyánszky, 2009; Jackson, Wolf, Johnston, Raymond, & Linden, 2008; Jackson, Wu, Linden, & Raymond, 2009; Langeslag, Morgan, Jackson, Linden, & Van Strien, 2009; Sessa, Luria, Gotler, Jolicœur, & Dell’Acqua, 2011), and fearful faces may be maintained using a higher resolution as compared with neutral faces (Sessa et al., 2011). Second, fearful faces may be more easily bound to their locations than neutral faces. Although no direct evidence has been provided to show that fearful faces are able to improve identity-location binding, several studies have reported that the spatial locations of negative emotional stimuli (e.g., words and pictures) are better remembered than neutral ones, which does indicate a better binding between the two (D’Argembeau & Van der Linden, 2004; Mather & Nesmith, 2008). Taken together, fearful target faces may facilitate identity tracking through the enhancement of maintenance of target identities and identity-location binding in visual working memory. The present finding that location tracking improved in addition to identity tracking is also consistent with the common resource model of MIT (Cohen et al., 2011; Pinto et al., 2010). Cohen et al. (2011) suggested that tracking the objects’ locations cannot be accomplished independently from tracking their identities; rather, they draw on a common resource that can be flexibly distributed between identity tracking and location tracking. Fearful expressions make identity-location binding easier, and therefore result in freeing up more mental resources that can be devoted to track the objects’ locations.

The present research demonstrates that facial expressions of distractors are not able to influence tracking performance. This may be related to a degree of distractor inhibition during the tracking process. Pylyshyn and colleagues (Flombaum, Scholl, & Pylyshyn, 2008; Pylyshyn, 2006; Pylyshyn, Haladjian, King, & Reilly, 2008) employed a probe detection task to explore the distribution of attention on targets, distractors and background during the tracking process and found that the detection rate was higher when the probe point emerged on either the targets or background, than when it emerged on the distractors. This suggests that distractors are inhibited during tracking. ERP data (Doran & Hoffman, 2010) has also demonstrated the existence of such a mechanism. Using faces with different identities as tracking stimuli, Ren et al. (2009) found that the identities of distractor faces are not processed. Likewise, Liu and Chen (2012) also found that the degree of attractiveness of distractor faces does not influence multiple face tracking performance. Collectively, these findings indicated that targets are processed more deeply than distractors during tracking, and that there is an inhibition effect of attention on distractors that inhibits the processing of distractor facial expressions.

In summary, the current research shows that facial expressions with abundant task-relevant social information can influence visual tracking performance. The advantage of tracking fearful target faces is only present when sufficient attentional resources are available, which suggests that the effect of fearful expressions on multiple face tracking is mediated by the availability of attentional resources. The finding that visual tracking is sensitive to the expression of target faces contrasts with visual index theory, which posits that tracking is achieved in early vision and completely encapsulated from high-level cognitive processes. Fearful faces may be maintained more efficiently in visual working memory and bound more easily to their locations than neutral ones. However, given that the present research only chose fearful faces as tracking stimuli, future studies should adopt other facial expressions (e.g., anger and happiness) in order to further investigate this issue.

## Competing Interests

The authors declare that they have no competing interests.

## Figures and Tables

**Figure 4:**
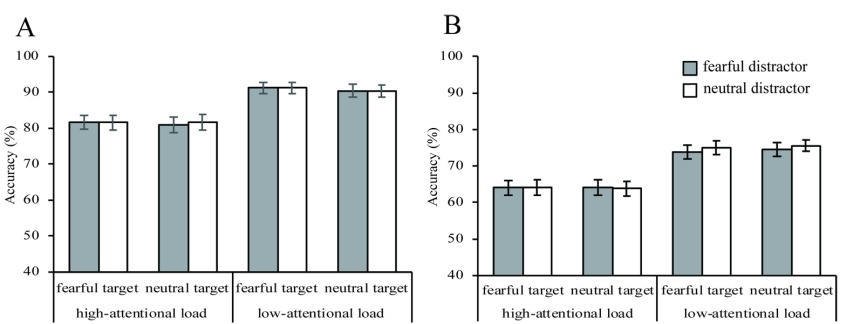
Data from control experiment. Mean tracking accuracy in low- and high-attentional load conditions as a function of target and distractor facial expressions. (A) Results for location tracking. (B) Results for identity tracking. Error bars represented ±1 standard errors.

## Footnotes

Development of the MacBrain Face Stimulus Set was overseen by Nim Tottenham and supported by the John D. and Catherine T. MacArthur Foundation Research Network on Early Experience and Brain Development. Please contact Nim Tottenham at tott0006@tc.umn.edu for more information concerning the stimulus set.

## Appendix

### Participants

A total of 13 observers (6 males; 18–25 years of age, mean = 21.08 ± 2.78 years) participated in the control experiment and received course credits as compensation. All participants reported having normal or corrected-to-normal vision and gave written informed consent prior to testing. The experiment was approved by the Research Ethics Board of Zhejiang University.

### Stimuli and Procedure

The stimuli and procedure were very similar to the main experiment, except that all the faces were inverted. It is well known that inversion disrupts facial processing (Tanaka & Farah, 1993; Yin, 1969) and the recognition of emotional expressions (Searcy & Bartlett, 1996; de Gelder, Teunisse, & Benson, 1997), while retaining low-level image differences. Therefore, we used inverted presentation to clarify whether the observed advantage in the tracking of fearful faces is attributable to simple low-level visual properties afforded to fearful faces or their emotional content. Equivalent results will be expected for upright and inverted displays if the tracking advantage of fearful faces is due to visual properties.

### Results

The results from control experiment are plotted in Figure 4. Performing a four-way within-subjects repeated measures ANOVA, we found location accuracy was significantly higher than identity accuracy, *F* (1, 12) = 197.230, *p* < .001, ŋ_p_^2^ = 0.943, and accuracy was higher when attentional load was low than when high, *F* (1, 12) = 146.540, *p* < .001, ŋ_p_^2^ = 0.924. However, no significant main effect was found for target facial expressions, *F* (1, 12) = 0.260, *p* = .619, ŋ_p_^2^ = 0.021, or distractor facial expressions, *F* (1, 12) = 0.680, *p* = .426, ŋ_p_^2^ = 0.054. What’s more, none of interactions was significant, *ps* > .05. Together, the results from control experiment showed that when inverted, fearful faces did not enjoy any tracking advantage over neutral faces, thus indicating that facial expressions influence visual tracking at a high computational level.

## References

[1] Allen R., Gabbert F. (2013). Exogenous social identity cues differentially affect the dynamic tracking of individual target faces. Journal of Experimental Psychology: Learning, Memory and Cognition.

[2] Allen R., Mcgeorge P., Pearson D. G., Milne A. (2006). Multiple-target tracking: A role for working memory?. The Quarterly journal of experimental psychology.

[3] Anderson A. K., Christoff K., Panitz D., De Rosa E., Gabrieli J. D. (2003). Neural correlates of the automatic processing of threat facial signals. The Journal of Neuroscience.

[4] Bae G. Y., Flombaum J. I. (2012). Close encounters of the distracting kind: Identifying the cause of visual tracking errors. Attention, Perception, & Psychophysics.

[5] Bankó É. M., Gál V., Vidnyánszky Z. (2009). Flawless visual short-term memory for facial emotional expressions. Journal of Vision.

[6] Botterill K., Allen R., McGeorge P. (2011). Multiple-object tracking: The binding of spatial location and featural identity. Experimental psychology.

[7] Carlson J. M., Reinke K. S. (2010). Spatial attention-related modulation of the N170 by backward masked fearful faces. Brain and Cognition.

[8] Carlson J. M., Reinke K. S., Habib R. (2009). A left amygdala mediated network for rapid orienting to masked fearful faces. Neuropsychologia.

[9] Cohen M. A., Pinto Y., Howe P. D. L., Horowitz T. S. (2011). The what-where trade-off in multiple-identity tracking. Attention, Perception, & Psychophysics.

[10] D’Argembeau A., Van der Linden M. (2004). Influence of affective meaning on memory for contextual information. Emotion.

[11] de Gelder B., Teunisse J. P., Benson P. J. (1997). Categorical perception of facial expressions: categories and their internal structure. Cognition and Emotion.

[12] de-Wit L. H., Lefevre C. E., Kentridge R. W., Rees G., Saygin A. P. (2011). Investigating the status of biological stimuli as objects of attention in multiple object tracking. PloS one.

[13] Dolan R. J., Vuilleumier P. (2003). Amygdala automaticity in emotional processing. Annals of the New York Academy of Sciences.

[14] Doran M. M., Hoffman J. E. (2010). The role of visual attention in multiple object tracking: Evidence from ERPs. Attention, Perception, & Psychophysics.

[15] Drew T., Vogel E. K. (2008). Neural measures of individual differences in selecting and tracking multiple moving objects. The Journal of Neuroscience.

[16] Eastwood J. D., Smilek D., Merikle P. M. (2001). Differential attentional guidance by unattended faces expressing positive and negative emotion. Perception & psychophysics.

[17] Eastwood J. D., Smilek D., Merikle P. M. (2003). Negative facial expression captures attention and disrupts performance. Perception & psychophysics.

[18] Eimer M., Holmes A., McGlone F. P. (2003). The role of spatial attention in the processing of facial expression: An ERP study of rapid brain responses to six basic emotions. Cognitive, Affective, & Behavioral Neuroscience.

[19] Eimer M., Kiss M. (2007). Attentional capture by task-irrelevant fearful faces is revealed by the N2pc component. Biological psychology.

[20] Esteves F., Dimberg U., Öhman A. (1994). Automatically elicited fear: Conditioned skin conductance responses to masked facial expressions. Cognition & Emotion.

[21] Flombaum J. I., Scholl B. J., Pylyshyn Z. W. (2008). Attentional resources in visual tracking through occlusion: The high-beams effect. Cognition.

[22] Fougnie D., Marois R. (2006). Distinct capacity limits for attention and working memory: Evidence from attentive tracking and visual working memory paradigms. Psychological Science.

[23] Fougnie D., Marois R. (2009). Attentive tracking disrupts feature binding in visual working memory. Visual Cognition.

[24] Fox E. (2002). Processing emotional facial expressions: The role of anxiety and awareness. Cognitive, Affective, & Behavioral Neuroscience.

[25] Franconeri S. L., Alvarez G. A., Enns J. T. (2007). How many locations can be selected at once?. Journal of Experimental Psychology: Human Perception and Performance.

[26] Franconeri S., Jonathan S., Scimeca J. (2010). Tracking multiple objects is limited only by object spacing, not by speed, time, or capacity. Psychological Science.

[27] Franconeri S. L., Lin J. Y., Enns J., Pylyshyn Z. W., Fisher B. (2008). Evidence against a speed limit in multiple-object tracking. Psychonomic bulletin & review.

[28] Frischen A., Eastwood J. D., Smilek D. (2008). Visual search for faces with emotional expressions. Psychological Bulletin.

[29] Globisch J., Hamm A. O., Esteves F., Öhman A. (1999). Fear appears fast: Temporal course of startle reflex potentiation in animal fearful subjects. Psychophysiology.

[30] Gray K. L., Adams W. J., Hedger N., Newton K. E., Garner M. (2013). Faces and awareness: Low-level, not emotional factors determine perceptual dominance. Emotion.

[31] Holmes A., Vuilleumier P., Eimer M. (2003). The processing of emotional facial expression is gated by spatial attention: evidence from event-related brain potentials. Cognitive Brain Research.

[32] Horowitz T. S., Klieger S. B., Fencsik D. E., Yang K. K., Alvarez G. A., Wolfe J. M. (2007). Tracking unique objects. Attention, Perception, & Psychophysics.

[33] Howe P. D., Holcombe A. O. (2012). The effect of visual distinctiveness on multiple object tracking performance. Frontiers in psychology.

[34] Howe P. D., Horowitz T. S., Morocz I. A., Wolfe J., Livingstone M. S. (2009). Using fMRI to distinguish components of the multiple object tracking task. Journal of Vision.

[35] Huang D., Zhang Y., Zhang K. (2014). The effects of the relationships between object features on multiple-identity tracking. Experimental psychology.

[36] Intriligator J., Cavanagh P. (2001). The spatial resolution of visual attention. Cognitive psychology.

[37] Iordanescu L., Grabowecky M., Suzuki S. (2009). Demand-based dynamic distribution of attention and monitoring of velocities during multiple-object tracking. Journal of Vision.

[38] Jackson M. C., Wolf C., Johnston S. J., Raymond J. E., Linden D. E. (2008). Neural correlates of enhanced visual short-term memory for angry faces: an FMRI study. PloS one.

[39] Jackson M. C., Wu C.-Y., Linden D. E., Raymond J. E. (2009). Enhanced visual short-term memory for angry faces. Journal of Experimental Psychology: Human Perception and Performance.

[40] Langeslag S. J., Morgan H. M., Jackson M. C., Linden D. E., Van Strien J. W. (2009). Electrophysiological correlates of improved short-term memory for emotional faces. Neuropsychologia.

[41] Liu C., Chen W. (2012). Beauty is better pursued: Effects of attractiveness in multiple-face tracking. the Quarterly journal of experimental psychology.

[42] Liu T., Chen W., Liu C. H., Fu X. (2012). Benefits and costs of uniqueness in multiple object tracking: The role of object complexity. Vision Research.

[43] Liu T., Chen W., Xuan Y., Fu X. (2009). The effect of object features on multiple object tracking and identification. Engineering Psychology and Cognitive Ergonomics: Lecture Notes in Computer Science.

[44] Makovski T., Jiang Y. V. (2009a). Feature binding in attentive tracking of distinct objects. Visual Cognition.

[45] Makovski T., Jiang Y. V. (2009b). The role of visual working memory in attentive tracking of unique objects. Journal of Experimental Psychology: Human Perception and Performance.

[46] Masterson F. A., Crawford M. (1982). The defense motivation system: A theory of avoidance behavior. Behavioral and brain sciences.

[47] Mather M., Nesmith K. (2008). Arousal-enhanced location memory for pictures. Journal of memory and language.

[48] Mineka S., Öhman A. (2002). Phobias and preparedness: The selective, automatic, and encapsulated nature of fear. Biological psychiatry.

[49] Öhman A. (2002). Automaticity and the amygdala: Nonconscious responses to emotional faces. Current directions in psychological science.

[50] Oksama L., Hyönä J. (2004). Is multiple object tracking carried out automatically by an early vision mechanism independent of higher-order cognition? An individual difference approach. Visual Cognition.

[51] Oksama L., Hyönä J. (2008). Dynamic binding of identity and location information: A serial model of multiple identity tracking. Cognitive psychology.

[52] Peirce J. W. (2007). PsychoPy – psychophysics software in Python. Journal of neuroscience methods.

[53] Peirce J. W. (2008). Generating stimuli for neuroscience using PsychoPy. Frontiers in neuroinformatics.

[54] Pessoa L., McKenna M., Gutierrez E., Ungerleider L. (2002). Neural processing of emotional faces requires attention. Proceedings of the National Academy of Sciences.

[55] Pessoa L., Padmala S., Morland T. (2005). Fate of unattended fearful faces in the amygdala is determined by both attentional resources and cognitive modulation. Neuroimage.

[56] Pinto Y., Howe P. D. L., Cohen M. A., Horowitz T. S. (2010). The more often you see an object, the easier it becomes to track it. Journal of Vision.

[57] Pourtois G., Grandjean D., Sander D., Vuilleumier P. (2004). Electrophysiological correlates of rapid spatial orienting towards fearful faces. Cerebral Cortex.

[58] Purcell D. G., Stewart A. L., Skov R. B. (1996). It takes a confounded face to pop out of a crowd. Perception.

[59] Pylyshyn Z. W. (1989). The role of location indexes in spatial perception: A sketch of the FINST spatial-index model. Cognition.

[60] Pylyshyn Z. W. (1994). Some primitive mechanisms of spatial attention. Cognition.

[61] Pylyshyn Z. W. (2001). Visual indexes, preconceptual objects, and situated vision. Cognition.

[62] Pylyshyn Z. W. (2006). Some puzzling findings in multiple object tracking (MOT): II. Inhibition of moving nontargets. Visual Cognition.

[63] Pylyshyn Z. W. (2007). Things and places: How the mind connects with the world.

[64] Pylyshyn Z. W., Haladjian H. H., King C. E., Reilly J. E. (2008). Selective nontarget inhibition in multiple object tracking. Visual Cognition.

[65] Pylyshyn Z. W., Storm R. W. (1988). Tracking multiple independent targets: Evidence for a parallel tracking mechanism. Spatial vision.

[66] Ren D., Chen W., Liu C. H., Fu X. (2009). Identity processing in multiple-face tracking. Journal of Vision.

[67] Scholl B. J., Pylyshyn Z. W. (1999). Tracking multiple items through occlusion: Clues to visual objecthood. Cognitive psychology.

[68] Scholl B. J., Pylyshyn Z. W., Feldman J. (2001). What is a visual object? Evidence from target merging in multiple object tracking. Cognition.

[69] Searcy J. H., Bartlett J. C. (1996). Inversion and processing of component and spatial-relational information in faces. Journal of Experimental Psychology: Human Perception and Performance.

[70] Sears C. R., Pylyshyn Z. W. (2000). Multiple object tracking and attentional processing. Canadian Journal of Experimental Psychology.

[71] Sessa P., Luria R., Gotler A., Jolicœur P., Dell’Acqua R. (2011). Interhemispheric ERP asymmetries over inferior parietal cortex reveal differential visual working memory maintenance for fearful versus neutral facial identities. Psychophysiology.

[72] Shim W. M., Alvarez G. A., Jiang Y. V. (2008). Spatial separation between targets constrains maintenance of attention on multiple objects. Psychonomic bulletin & review.

[73] Tanaka J. W., Farah M. J. (1993). Parts and wholes in face recognition. Quarterly Journal of Experimental Psychology.

[74] Taylor T. L., Therrien M. E. (2005). Inhibition of return for faces. Perception & psychophysics.

[75] Tombu M., Seiffert A. E. (2011). Tracking planets and moons: Mechanisms of object tracking revealed with a new paradigm. Attention, Perception, & Psychophysics.

[76] Trick L. M., Mutreja R., Hunt K. (2012). Spatial and visuospatial working memory tests predict performance in classic multiple-object tracking in young adults, but nonspatial measures of the executive do not. Attention, Perception, & Psychophysics.

[77] Van Kleef G. A. (2009). How emotions regulate social life the emotions as social information (EASI) model. Current directions in psychological science.

[78] Vuilleumier P., Armony J. L., Driver J., Dolan R. J. (2001). Effects of attention and emotion on face processing in the human brain: an event-related fMRI study. Neuron.

[79] Yang E., Blake R. (2012). Deconstructing continuous flash suppression. Journal of Vision.

[80] Yantis S. (1992). Multielement visual tracking: Attention and perceptual organization. Cognitive psychology.

[81] Yin R. K. (1969). Looking at upside-down faces. Journal of Experimental Psychology.

